# Identification and structural analysis of the *Schizosaccharomyces pombe* SMN complex

**DOI:** 10.1093/nar/gkab158

**Published:** 2021-03-23

**Authors:** Jyotishman Veepaschit, Aravindan Viswanathan, Rémy Bordonné, Clemens Grimm, Utz Fischer

**Affiliations:** Department of Biochemistry, Biocenter, University of Würzburg, Würzburg 97074, Germany; Department of Biochemistry, Biocenter, University of Würzburg, Würzburg 97074, Germany; Institut de Génétique Moléculaire de Montpellier, University of Montpellier, CNRS, Montpellier 34293, France; Department of Biochemistry, Biocenter, University of Würzburg, Würzburg 97074, Germany; Department of Biochemistry, Biocenter, University of Würzburg, Würzburg 97074, Germany

## Abstract

The macromolecular SMN complex facilitates the formation of Sm-class ribonucleoproteins involved in mRNA processing (UsnRNPs). While biochemical studies have revealed key activities of the SMN complex, its structural investigation is lagging behind. Here we report on the identification and structural determination of the SMN complex from the lower eukaryote *Schizosaccharomyces pombe*, consisting of SMN, Gemin2, 6, 7, 8 and Sm proteins. The core of the SMN complex is formed by several copies of SMN tethered through its C-terminal alpha-helices arranged with alternating polarity. This creates a central platform onto which Gemin8 binds and recruits Gemins 6 and 7. The N-terminal parts of the SMN molecules extrude via flexible linkers from the core and enable binding of Gemin2 and Sm proteins. Our data identify the SMN complex as a multivalent hub where Sm proteins are collected in its periphery to allow their joining with UsnRNA.

## INTRODUCTION

UsnRNPs constitute the central building blocks of major and minor spliceosomes, which catalyze pre-messenger RNA (pre-mRNAs) splicing (1,2). In higher eukaryotes roughly 2–5 × 10^6^ UsnRNPs accumulate in the nucleus of a given cell to ensure splicing of all cellular mRNAs (3). This demands for a highly efficient and regulated production line that encompasses nucleo-cytoplasmic transport processes as well as the aid of a specific set of assembly factors (4–6). The RNA moieties of UsnRNPs are transcribed by either polymerase II (U1, U2, U4, U5, U11, U12 and U4atac snRNAs) or polymerase III (U6 and U6atac snRNAs). The former snRNAs are transiently exported to the cytoplasm to assemble with seven Sm proteins (SmB/B’, SmD1, SmD2, SmD3, SmE, SmF and SmG). This results in the formation of the toroidal Sm core, which is a common structural denominator of these UsnRNPs (7–10). 5′ cap trimethylation and nuclear import of the assembled UsnRNPs concludes the cytosolic maturation phase (11–14). Biogenesis of UsnRNPs is completed in Cajal bodies, where specific proteins are recruited and UsnRNAs become modified (15–17).

The cytosolic assembly phase of UsnRNPs is aided by the Protein Arginine Methyltransferase 5 (PRMT5) complex acting together with the Survival Motor Neuron (SMN) complex (4,5,18,19). The PRMT5 complex consists of the methyltransferase PRMT5, the assembly chaperone pICln and WD45 (also termed MEP50) and acts early in the assembly pathway. Its main task is to catalyze symmetric methylation of arginine residues in Sm proteins and the formation of higher order Sm protein complexes (20–23). For this, the assembly chaperone pICln recruits all newly synthesized Sm proteins to the PRMT5 complex (24). This leads to the formation of two different assembly intermediates: a ring-shaped 6S complex composed of pICln and SmD1, SmD2, SmE, SmF and SmG and a pICln-SmB-SmD3 heterotrimer (25,26). Because association of pICln with Sm proteins prevents binding onto UsnRNA, the activity of additional factors united in the SMN complex is required (25–29). In vertebrates this macromolecular machinery consists of nine factors, including the survival motor neuron (SMN) protein, Gemins2-8 (abbreviated G2-8 with prefix Hs for human and Sp for *Schizosaccharomyces pombe* throughout the paper) and unrip (27–33). While SMN and G2 engage with the Sm proteins and aid in the release of pICln, G5 has been reported to be the snRNA recruiter during UsnRNP assembly (29,34–37).

Consistent with its reported role in RNP biogenesis, several factors of the assembly machinery including SMN have been shown to be essential for viability (38–40). Interestingly, the human disorder spinal muscular atrophy (SMA) is causally linked to reduced levels of functional SMN. SMN deficiency alters the stoichiometry of snRNAs in SMN-deficient mouse tissues and causes widespread and tissue-specific pre-mRNA splicing defects in SMA animal models. SMA might hence arise from the inefficient splicing of pre-mRNAs coding for proteins required for motor neuron function (41–43).

Biochemical and genetic studies enabled insight into the role of the SMN complex in UsnRNP assembly. Structural insight into the architecture of the SMN complex, however, is still limited. Thus far, SMN’s Tudor domain (44) and C-terminal region (45,46), the WD-repeat domain of G5 (35,36) and parts of a G6/G7 dimer (47) have been determined by X-ray crystallography or NMR studies. In addition, assembly intermediates encompassing the N-terminus of SMN bound to G2 and Sm proteins have been structurally analyzed (29,34), which provided important insight into the mechanism of pICln release and Sm protein arrangements on the complex.

In this paper, we describe the identification of a simplified version of the SMN complex in the fission yeast *Schizosaccharomyces pombe* consisting of five proteins only. The biochemical reconstitution of the yeast SMN (SpSMN) complex allowed us to determine its structure by a combination of X-ray crystallography, homology modeling, and small angle X-ray scattering (SAXS) analysis. These studies identified the SMN complex as a multivalent hub where Sm proteins are collected in its periphery to allow their joining with UsnRNA.

## MATERIALS AND METHODS

### Plasmid construction: *S. pombe*

Genes encoding SpSMN complex components (and variants thereof) were first cloned as mono-cistronic constructs into either pETM-11 (N-terminal His_6_-tag) or pETM-13 (No tags) vectors. Following this, various polycistronic constructs were generated by iterative cloning using the isocaudomers XbaI and NheI restriction sites, in a strategy similar to what has been previously described (25). For the determination of the interaction map and *in vitro* reconstitution of the SpSMN complex, polycistronic constructs of full-length proteins were designed as SpSMN/His_6_-SpG2, His_6_-SpSMN/SpG2/SpG8, SpG6/SpG7/His_6_-SpG8, and SpG6/His_6_-SpG7. To investigate the interaction between SpG8 and SpSMN, the constructs His_6_-SpSMN^ΔYG^/SpG2/SpG8 (YG = residues 130–152) and SpG6/SpG7/His_6_-SpG8^ΔN58^ were designed. For crystallization and SAXS experiments the constructs SpSMN^Δ36–119^/His_6_-SpG2^ΔN80^, SpSMN/His_6_-SpG2^ΔN80^, and SpG6/SpG7/His_6_-SpG8^Δ35–58^ were designed. For mutational analysis, constructs SpSMN^Δ36–119^S130D/His_6_-SpG2^ΔN80^ and SpSMN^Δ36–119^A134E/His_6_-SpG2^ΔN80^ were designed. Each poly-cistronic construct was designed under a single T7 promotor and individual ribosome binding sites for each gene.

### Plasmid construction: human

Genes encoding human SMN complex components (and variants thereof) were sub-cloned into either pETM-30 (N-terminal His_6_-GST-tag), pETM-11 (N-terminal His_6_-tag) or pETM-13 (No tags) from DNA plasmids described previously (1). Truncation variants were generated with specific primers and mutants were generated by overlap extension PCR. Poly-cistronic plasmids were generated by iterative cloning employing the isocaudomers XbaI and NheI, similar to a strategy described previously (25). For crystallization, the constructs His_6_-GST-HsG8^190–230^ and HsG6^1–92^/His_6_-HsG7^46–131^ were designed. Similar to *S. pombe* constructs, each poly-cistronic construct was designed under a single T7 promotor and individual ribosome binding sites for each gene. MBP fusion proteins of human YG-box^252–284^ constructs (and variants thereof) were designed using the pETM-41 vector. All pETM vectors were obtained from EMBL protein expression facility (Heidelberg, Germany).

### Protein expression in *E. coli*

Recombinant proteins and/or protein complexes were produced either by single expression of plasmids or by co-expression from poly-cistronic constructs using BL21(DE3) competent cells (NEB #C2527I). Transformed bacterial cells were cultured in TB medium containing 1× TB buffer (17 mM KH_2_PO_4_; 72 mM K_2_HPO_4_), 2 mM MgCl_2_, and appropriate antibiotics until OD_600_ of 1.0 at 37°C and 215 rpm. Then, protein expression was induced by adding 0.5 mM IPTG and the cultures were left to grow for 18 h at 15°C and 215 rpm. Cells were harvested by centrifugation and cell pellets resuspended in either lysis-buffer1 (150 mM NaCl; 50 mM HEPES pH 7.4; 20 mM Imidazole; 2 mM 2-mercaptoethanol; 10% glycerol) for *S. pombe* proteins or in lysis-buffer2 (200 mM NaCl; 50 mM HEPES pH 7.0; 25 mM Imidazole; 5 mM 2-mercaptoethanol) for human proteins, each containing protease inhibitors. Cell suspensions were snap frozen in liquid nitrogen and stored at –20°C until further use.

### Protein purification

Frozen cell suspensions were thawed and subsequently lysed by sonication (Branson Sonifier 250). Lysed cell suspension was clarified by centrifugation at 30 000 rpm (rotor 45 Ti, Beckman Coulter) at 4°C for 1 h. Cleared lysate was incubated with Ni-NTA agarose beads (Qiagen) or Glutathione Sepharose 4B (GE Healthcare) for 2 h at 4°C. Following this, the beads were washed with 20–40 bed volumes of lysis buffer1/2 and the bound proteins were eluted with 250 mM Imidazole or 20 mM GSH. The eluted proteins were supplemented with 1–2% (w/w) TEV protease (for His_6_ removal) or PreScission™ protease (for His_6_-GST removal). *S. pombe* proteins were subsequently dialyzed into gel filtration buffer (150 mM NaCl; 20 mM HEPES pH 7.4; 2 mM DTT) overnight at 4°C and the dialysate was concentrated for further steps. HsG8^190–230^ was subsequently incubated with Ni-NTA beads and HsG6^1–92^/His_6_-HsG7^46–131^ was used as bait to purify the trimeric complex.

### Gel filtration and *in vitro* reconstitution

Purified complexes were further characterized using analytical gel filtration columns Superose 6 10/300, Superdex 75 10/300, and Superdex 200 10/300 (GE Healthcare, Munich, Germany). For reconstitution assays, equimolar amounts of SpSMN sub-complexes were combined and incubated on ice for 15 min. Hereafter, the samples were briefly placed on 37°C for 5 min followed by an additional 15 min on ice. The samples were then centrifuged at 10 000 g for 15 min at 4°C before applying onto gel filtration columns. Gel filtration fractions were analyzed by 15% Tris–tricine SDS-PAGE.

### Crystallization and structure determination

Crystallization trials were conducted with the SpSMN^Δ36–119^/SpG2^ΔN80^ complex at a concentration of 19.7 mg/ml. Needle shaped crystals of 0.6 mm size of space group *C*2 2 2_1_ were obtained with a condition containing 65% 2-methylpentanediol, 80 mM KCl, and 40 mM HEPES (Natrix HT crystallization screen, Hampton Research) at different pH values (6.8, 6.9, and 7.2), by the hanging drop vapor diffusion method. The crystals were snap frozen in liquid nitrogen in the mother liquor and X-ray diffraction data were collected. Phases were determined by molecular replacement using the dimeric SpYG-domain structure (PDB ID: 4RG5 (46)) as a template. Electron density for the globular SpG2^ΔN80^ could not be assigned. Instead, electron density for helical dimers of SpSMN^Δ36–119^ was clearly observed. The absence of SpG2^ΔN80^ from the crystals is attributed to the denaturation of this compound.

HsG6^1–92^/HsG7^46–131^/HsG8^191–230^ crystals were grown at a concentration of 30 mg/mL in 100 mM 2-(*N*-morpholino) ethanesulfonic acid, 200 mM NaCl and 30% Jeffamine ED2003 by sitting-drop vapour diffusion at 18°C. Crystals were transferred into a cryoprotectant solution containing 100 mM 2-(*N*-morpholino) ethanesulfonic acid, 200 mM NaCl and 35% Jeffamine ED2003 before being snap frozen in liquid nitrogen. The structure of this trimeric complex was solved using the HsG6/HsG6 (PDB ID: 1Y96 (47)) dimer as the molecular replacement model and the HsG8^190–230^ fragment could be traced from the initial 2*F*_o_ – *F*_c_ density map. The resulting model could be refined to an R_free_/R_work_ of 0.205/0.250 and included residues 1–86, residues 47–131 and residues 191–227 for HsG6, HsG7 and HsG8 respectively.

The data sets for each of the protein crystals were collected at the ID30B beam line of the European Synchrotron Radiation Facility (ESRF, Grenoble, France) and processed with XDS (48). The structures were solved by molecular replacement with PHASER (49). Automated refinement was performed in PHENIX until R/R_free_ factors converged. The crystallographic data processing and refinement parameters are summarized in Table 1. The final Figures were generated using PyMOL Molecular Graphics System, Version 2.0, Schrödinger, LLC.

**Table 1.** Crystallographic data and refinement statistics

Parameter	HsG^61–92^/HsG7^46–131^/HsG8^191–230^	SpSMN^Δ36–119^
Wavelength (Å)	0.9762	0.9762
Resolution range (Å)	48.07–1.52 (1.57–1.52)	40.49–2.15 (2.23–2.15)
Space group	*P*22_1_2_1_	*C*222_1_
Unit cell	*a* = 59.88, *b* = 80.59, *c* = 82.66; α = 90, β = 90, γ = 90	*a* = 27.19, *b* = 83.71, *c* = 160.06; α = 90, β = 90, γ = 90
Total reflections	223044 (10189)	67776 (7057)
Unique reflections	58263 (4340)	10282 (1010)
Multiplicity	3.8 (2.3)	6.6 (7.0)
Completeness (%)	93.5 (70.8)	99.3 (99.7)
Mean I/sigma(I)	10.83 (1.03)	10.85 (2.25)
Wilson *B*-factor (Å^2^)	18.6	46.4
*R*_meas_ (%)^a^	7.6 (95.9)	11.4 (89.0)
CC1/2 (%)	99.9 (19.7)	99.8 (70.8)
Reflections used in refinement	58 076 (4340)	10 262 (1010)
Reflections used for *R*-free	2873 (207)	531 (31)
*R*_work_ (%)^b^	20.5 (40.9)	25.6 (38.0)
*R*_free_ (%)^c^	25.0 (41.9)	29.1 (37.8)
No. of non-hydrogen atoms	3735	927
Ligand	–	32 (MPD)
Water	363	3
No. of protein residues	418	108
RMSD^d^ bond lengths (Å)	0.010	0.004
RMSD^d^ bond angles (°)	1.020	0.800
Ramachandran favored (%)^e^	95.8	100
Ramachandran allowed (%)^e^	4.2	0.0
Ramachandran outliers (%)^e^	0.0	0.0
Rotamer outliers (%)^e^	0.6	0.0
Clash score^e^	1.64	1.10
Average *B*-factor (Å^2^)	27.4	69.8
Macromolecules	26.6	69.8
Waters	34.4	54.9
Ligands	-	73
PDB code	7BBL	7BB3

^a^ *R*_meas_ = Σ_h_(n/n – 1)^1/2^Σ_i_|I_i_(h) – <I(h)>|/Σ_h_Σ_i_I_i_(h), where I_i_(h) and <I(h)> are the ith and mean measurement of the intensity of reflection h.

^b^ *R*_work_ = Σ_h_||F_obs_(h)| – |F_calc_(h)||/Σ_h_|F_obs_(h)|, where F_obs_(h) and F_calc_(h) are the observed and calculated structure factors, respectively.

^c^ *R*_free_ is the R-value obtained for a test set of reflections consisting of a randomly selected 5% subset of the data set excluded from refinement.

^d^Root Mean Square Deviation

^e^Values from Molprobity server (http://molprobity.biochem.duke.edu/)

Values in parenthesis are for the highest resolution shell.

### *In vitro* transcription and translation of human Gemin8

N-terminal His_6_-HsG8 (full length) was *in vitro* transcribed and translated with [^35^S]-Methionine labeling with the TNT^®^ T7 Quick coupled Transcription/Translation system (Promega).

### *In vitro* protein binding assays

For the MBP binding assays, MBP fusion proteins immobilized on Amylose resin (NEB) were incubated with *in vitro* transcribed translated [^35^S]-methionine labeled Gemin8 transcripts in binding buffer (HEPES, pH 7.0, 150 mM NaCl, 2 mM DTT and protease inhibitors) at 4°C for 3 h. The resin was then washed initially with a high salt buffer (HEPES, pH 7.0, 300 mM NaCl, 2 mM DTT and protease inhibitors) followed by washes with the binding buffer. Bound proteins were then eluted with 1× SDS sample buffer, resolved by SDS-PAGE (13% Bis–Tris) and analyzed by Coomassie staining. Labeled proteins were detected by autoradiography of the dried gel.

### Yeast strains, media and genetic methods

Standard methods were used for growth and genetic manipulation of *S. pombe* (50). Cells were grown on YES or minimal EMM2 medium with adequate supplements. Strains carrying null allele of SpG6 (SPAC4D7.15::NatN2) and SpG7 (SPBC32F12.16::NatN2) were constructed in diploid strain (*h+/h+ ade6-M210/ade6-M216 ura4-D18/ura4-D18 leu1–32/leu1–32)* by homologous recombination as described previously using appropriate templates and primers (51). The diploid strain heterozygous for the null allele of SpG8 (SPBC16H5.15) (*h+/h+ ade6-M210/ade6-M216 ura4-D18/ura4-D18 leu1–32/leu1–32* SPBC16H5.15*/* SPBC16H5.15*::KanMX4*) was purchased from Bioneer Corporation (Korea). After transformation with the sporulation-inducing plasmid pON177 (52), spores were dissected and germinated at 25°C on YES plates. The *temperature-degron tdGemin8* allele was constructed using the pSMRG2-nmt41-degron plasmid (53) as described previously (54). A DNA fragment carrying 400 nucleotides homologies to genomic DNA was amplified and transformed into fission yeast wild-type cells. Correct homologous recombinations of the disrupted and tagged alleles were checked by PCR amplification of genomic DNA.

### Plasmid constructions

PCR fragments containing the coding sequences of the *S. pombe* Gemins were PCR amplified from genomic DNA or from the pTN-RC5 cDNA library (a gift from T. Nakamura, YGRC, Osaka, Japan) using forward and reverse oligonucleotides carrying adequate restriction sites. After separation on agarose gels, DNA fragments were purified using the GeneClean procedure and ligated into previously cut pREP41/42 or pREP41/42-GFP-N vector (55). The pASΔΔ and pACT2st vectors were used to constructs baits and preys for two-hybrid analyses (56). PCR amplification were performed from pREP plasmids containing the corresponding genes. Primer sequences and PCR regimes are available upon request. Construction of the SMN-A134E and SMN-S130D mutants was achieved using the QuikChange Site-Directed Mutagenesis kit (Stratagene, La Jolla CA, USA) essentially according to the manufacturer's instructions. All the cloning junctions and coding sequences were verified by sequencing.

### Two-hybrid assays for protein–protein interactions

Two-hybrid assays were performed with the CG1945 and Y187 strains (57). The CG1945 strain was transformed with the pASΔΔ– constructs and selected on –Trp plates while Y187 was transformed with the pACT2st- constructs and selected on –Leu plates. Strains carrying bait and prey plasmids were mated overnight on rich YPD plates and diploids containing the bait and prey combinations were selected on –Trp –Leu plates. Diploid yeast cells carrying bait/prey combinations were cultured in –Trp–Leu media and interactions were screened by spotting serial dilutions on –Trp–Leu–His plates. Incubations were performed at 30°C for 3–5 days.

### Purification of endogenous SpSMN complex

Yeast cells carrying a GFP-SpG6 fusion sequence were grown in EMM2 -Ura media to an OD_A600_ of 0.6–0.8 and the cell pellet was resuspended in lysis buffer (10 mM Tris/Cl pH 7.5; 150 mM NaCl; 0.5 mM EDTA; 0.25% NP-40; 1 mM PMSF; 1× Complete protease inhibitors) and frozen. For purification of the endogenous SpSMN complex, frozen cells were ground to fine powder using a Freezer Mill 6770 grounder (Spex) and after centrifugation at 14 000 rpm for 30 min, the soluble extract was recovered by centrifugation at 49 000 rpm for 1 h at 4°C and incubated with GFP-Trap beads (Chromotek, Germany) for 4 h at 4°C. The beads were then washed four times in wash buffer (10 mM Tris/Cl pH 7.5; 150 mM NaCl; 0.5 mM EDTA) and the immunoprecipitated proteins were separated by SDS-PAGE.

### Northern blot, primer extension and native gel electrophoresis

Total yeast RNA was purified from exponentially growing cells with Tri-Reagent (Sigma) according to the manufacturer's procedure. Primer extension and Northern blot analyses were performed as described previously (58). For native gel analysis of snRNPs, extracts were prepared from cells which were resuspended to 1 g/ml in AGK400 buffer (10 mM HEPES–KOH pH 7.9, 400 mM KCl, 1.5 mM MgCl_2_, 0.5 mM DTT, 1× Complete protease inhibitors and 10% glycerol). After freezing in liquid nitrogen, cells were ground to fine powder. After thawing on ice, cells were centrifuged at 14 000 rpm for 10 min at 4°C and the supernatant recovered and spun at 55 000 rpm for 30 min at 4°C in a TLA-100.3 rotor. The extract was then dialyzed for 2 h against buffer D (20 mM HEPES–KOH pH 7.9, 0.2 mM EDTA, 100 mM KCl, 0.5 mM DTT, 1 mM PMSF, 20% glycerol) and aliquots stored at –80°C. Native gel electrophoresis and analysis were performed as previously described (54).

### Small angle X-ray scattering data acquisition

Synchrotron SAXS data from solutions of protein complexes in 150 mM NaCl, 20 mM HEPES, 1 mM DTT, pH 7.5, were collected at the BM29 beam line of the European Synchrotron Radiation Facility (ESRF, Grenoble, France) using a PILATUS 1M detector (Dectris) at a distance of 2.867 m from the sample, and a wavelength of 0.9919 Å (*I*(s) versus *s*, where *s* = 4πsin θ/λ, and 2θ is scattering angle). Data collection was done for a scattering vector (*s*) range of 0.0032–0.4944 Å^−1^. In-line size-exclusion chromatography (SEC) was employed for the data collection. Protein solutions were injected onto a Superdex 200 10/300 column (GE Healthcare, Munich, Germany) at 20°C and run at a flowrate of 1 ml/min. A total of 1800 frames spanning the whole elution profile (with 1 s exposure per frame) were collected. The data was then normalized to the intensity of the transmitted beam and radially averaged. (see also Supplemetary Table S4).

### Small angle X-ray scattering data validation and analysis

All data processing was performed using ATSAS 3.0.3 software package (59). For data shown in Figure 7, 20 frames at the peak of the SEC-SAXS chromatogram were scaled and averaged. Background subtraction was performed using scaled and averaged buffer frames preceding the protein peak. Protein concentrations were obtained from the peak of the UV_280_ trace. For data shown in Supplementary Table S3, individual frames at various regions of the chromatograms were selected and background subtraction was performed using buffer frames. Protein concentrations at each selected frame was obtained from the UV_280_ trace of the chromatogram. Quality of each of the final scattering curve was investigated using Guinier plots (60). The radius of gyration (*R*_g_) was obtained from Guinier approximation: *I*(*s*) = *I*(0) exp(*s*^2^*R*_g_^2^/3), with the limits *sR*_g_ < 1.3. The pairwise distance distribution function *P*(*r*) and maximum particle dimension *D*_max_ were obtained from the GNOM program (61) integrated into the ATSAS software package. The molecular weights calculated from *I*(0) in Figure 7 and Supplementary Table S3 were obtained by the following formula: (MW^u^/MW^s^) = [I(0)^u^/Conc.^u^]/ [I(0)^s^/Conc.^s^], where u = unknown and s = standard. SAXS data of SpSMN^Δ36–119^S130D/SpG2^ΔN80^ was used as a standard. The molecular weight obtained from the Porod volume (*V*_p_) was calculated by the following formula: MW = *V*_p_/1.66.

## RESULTS

### Identification of the fission yeast SMN complex

**Figure 1.**
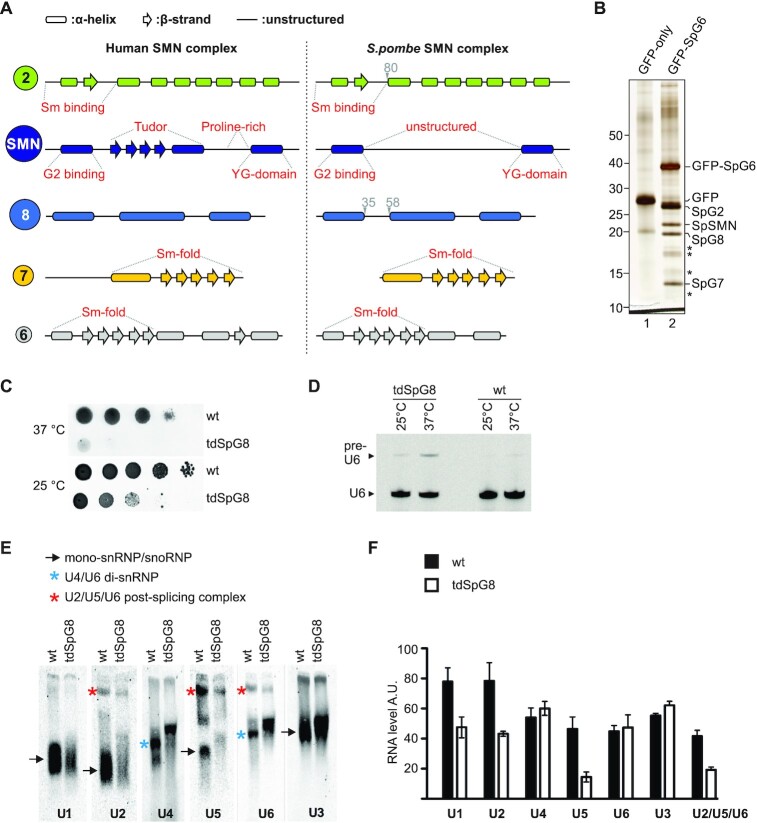
Identification of a pentameric SpSMN complex linked to UsnRNP assembly. (**A**) Secondary structural elements of the human and the *S. pombe* SMN complex core subunits (SMN, G2, G6, G7 and G8). Known domain compositions for the subunits are indicated. (**B**) Immunoprecipitation of endogenous SpSMN complex using GFP-Trap-A beads from cells expressing GFP-SpG6 as the sole source of SpG6 (lane 2). A control purification with GFP only is shown in lane 1 (4–12% gradient gel). The asterisks point to Sm proteins. (**C**) Serial dilutions of wild type and tdSpG8 cells were spotted onto rich media and grown at the indicated temperature. (**D**) Splicing inhibition in the tdSpG8 mutant. After growth of wild type cells and cells carrying the tdSpG8 allele at the indicated temperature for 4 h, total RNA was isolated and used for primer extension. Pre-U6 indicates the species corresponding to the U6 precursor and U6 indicates the spliced matured U6 RNA. (**E**) Native gel analysis of snRNPs in tdSpG8 and wild type cells. Extracts were prepared from cells grown at 25°C and similar amounts were separated on 4% native gels. The RNAs were subjected to Northern analysis and hybridized with oligonucleotide probes for the different snRNAs. The arrows indicate U1, U2, U5 (snRNPs) or U3 (snoRNP). Blue and red asterisks point to U4/U6 di-snRNPs and U2/U5/U6 post-splicing complexes, respectively. (**F**) quantification of snRNP levels using ImageJ. Data from two independent experiments are presented as mean ± SEM. A.U.: arbitrary units.

Only SMN and G2 orthologues of the human SMN complex have been found thus far in *S. pombe* (62–64). Using a bioinformatics approach, we identified putative orthologs of G6 (SpG6), G7 (SpG7) and G8 (SpG8) based on homology at the level of amino acid sequence and secondary structure (Figure 1A and Supplementary Figure S1). Whereas the sequence conservation of all three candidates is weak, their predicted secondary structures correspond well to their human counterparts. To investigate whether these factors are part of a larger complex, immunoprecipitation experiments were performed using extracts from strains expressing either GFP alone or GFP-tagged SpG6 as the sole source of SpG6. As determined by mass spectrometry, the immunoprecipitate contained apart from the tagged SpG6 bait, SpSMN, SpG2 as well as the newly identified orthologues SpG7 and SpG8 (Figure 1B and Supplementary Table S1). Importantly, Sm proteins were also found in this immunoprecipitation albeit in sub-stoichiometric amounts (Figure 1B (asterisks) and Supplementary Table S1). These findings show that the SpSMN complex consists of SpSMN, SpG2, SpG6, SpG7 and SpG8, and binds to Sm protein substrates. However, orthologues of HsG3–5 and unrip are lacking.

We next tested whether the SpSMN complex is functionally related to its human counterpart. Consistent with a role in UsnRNP assembly, a tetrad analyses showed that deletion of the SpG6–8 genes causes lethality (Supplementary Figure S2A), as has been shown already for SpSMN and SpG2, demonstrating that SpG6, SpG7 and SpG8 are essential genes. Furthermore, a yeast strain carrying a temperature-degron allele of SpG8 (tdSpG8) displays already a growth defect at permissive-temperature as well as reduced splicing after a shift to non-permissive temperature (Figure 1C and D). Lastly, extracts prepared from tdSpG8 cells contained decreased levels of the U1, U2 and U5 Sm-class snRNPs while the amount of the U3snoRNP (an RNP lacking Sm proteins) remained unaffected (black arrows, Figure 1E). Of note, the mobility of the U4/U6 di-snRNP is slightly decreased (blue asterisks, Figure 1E) and the amount of the post-splicing U2/U5/U6 complexes (red asterisks, Figure 1E) is decreased in the mutant, which indicates defects in spliceosome activity (see also Figure 1F for quantification of snRNP levels). Together, our data suggest that the SpSMN complex is required for formation of Sm-class UsnRNPs and splicing.

### Architecture and *in vitro* reconstitution of the SpSMN pentameric complex

**Figure 2.**
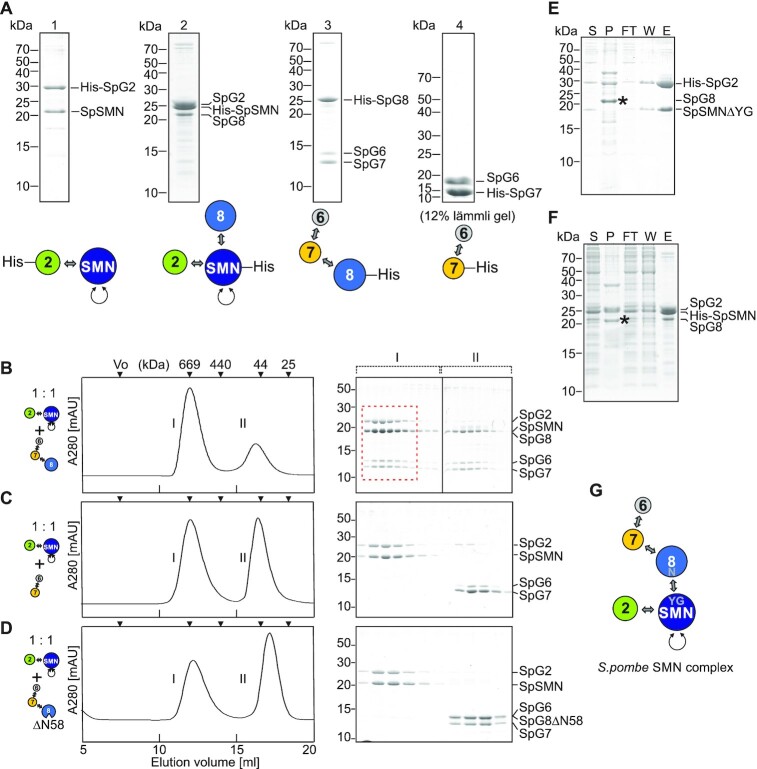
*In vitro* reconstitution of the pentameric SpSMN complex. (**A**) Recombinant co-expression and Ni-NTA purifications of SpSMN/His_6_-SpG2 (lane 1), His_6_-SpSMN/SpG2/SpG8 (lane 2), SpG6/SpG7/His_6_-SpG8/ (lane 3), and SpG6/His_6_-SpG7 (lane 4) sub-complexes from *E. coli*. Purified complexes were analyzed by 15% Tris-Tricine SDS-PAGE unless otherwise mentioned. (**B–D**) Complexation assays. SpSMN/SpG2 was added to equimolar amounts of SpG6/SpG7/SpG8 (B), SpG6/SpG7 (C), or SpG6/SpG7/SpG8ΔN58 (D), and the resulting mixtures analyzed by gelfiltration (Superose 6 10/300). Fractions under peaks I and II are analyzed by 15% Tris-Tricine SDS-PAGE. The formation of pentameric SpSMN complex is indicated by red dashed box in (B). (**E**) Ni-NTA purification of SpSMNΔYG/His_6_-SpG2/SpG8. YG refers to residues 130–152. (**F**) Ni-NTA purification of His_6_-SpSMN/SpG2/SpG8. Asterisk indicates insoluble SpG8. S = supernatant, P = pellet, FT = flow through, W = wash, E = elution. (**G**) Interaction map of the SpSMN complex.

The discovery of a simplified SMN complex in *S. pombe* enabled its biochemical and structural investigation. Earlier studies revealed an elaborate interaction network that ties together the proteins of the human SMN complex (31). In this network, HsSMN forms the central core onto which HsG2 binds via the N-terminus of HsSMN. The C-terminus of HsSMN, termed the YG-domain, establishes the connection to HsG8, which in turn recruits the HsG6/HsG7 heterodimer. In support of a similar protein network in the *S. pombe* complex we detected identical interaction pattern among the yeast orthologues using yeast two-hybrid assays (Supplementary Figure S2B). Furthermore, we succeeded in the co-expression and purification of SpSMN/SpG2, SpSMN/SpG2/SpG8, SpG6/SpG7/SpG8/ and SpG6/SpG7, providing biochemical evidence for the interaction network (Figure 2A and Supplementary Figure S3).

The availability of these protein modules enabled the reconstitution of the pentameric SpSMN complex *in vitro*. Equimolar amounts of bacterially expressed SpSMN/SpG2 and SpG6/SpG7/SpG8 complexes were mixed and subjected to gel filtration chromatography. All proteins elute in a single peak near the 669 kDa marker, showing the formation of the pentameric complex (red dashed box, Figure 2B). In the absence of SpG8, however, SpSMN/SpG2 and SpG6/SpG7 fail to form a complex and are completely separated into two distinct peaks (Figure 2C). Interestingly, a trimeric complex lacking the first 58 residues of SpG8 (SpG6/SpG7/SpG8ΔN58) also fails to form the pentameric SpSMN complex (Figure 2D). Furthermore, full-length SpG8 bound to SpSMN/SpG2 only when the YG-domain of SpSMN was present (Figure 2E and F). Thus, SpG8 forms the link between the SpSMN/SpG2 and SpG6/SpG7 dimers through an interaction of the N-terminus of SpG8 with the YG-domain of SpSMN (Figure 2G).

We noted that the hydrodynamic size of the SpSMN/SpG2 unit is almost identical to the size of the entire pentameric SpSMN complex with elution peaks at approx. 669 kDa on gel filtration columns (Figure 2B–D). In fact, no significant variation in its hydrodynamic size was observed when individual SpGemins or subunits thereof were bound onto the SpSMN/SpG2 module (Supplementary Figure S4). However, deletion of the YG-domain or the long unstructured region (residues 36–119) of SpSMN showed a drastic decrease in size (Supplementary Figure S4). Thus, the hydrodynamic properties of the whole SpSMN complex are primarily a function of the core SpSMN subunit.

### Structure of the G6/G7/G8 module

**Figure 3.**
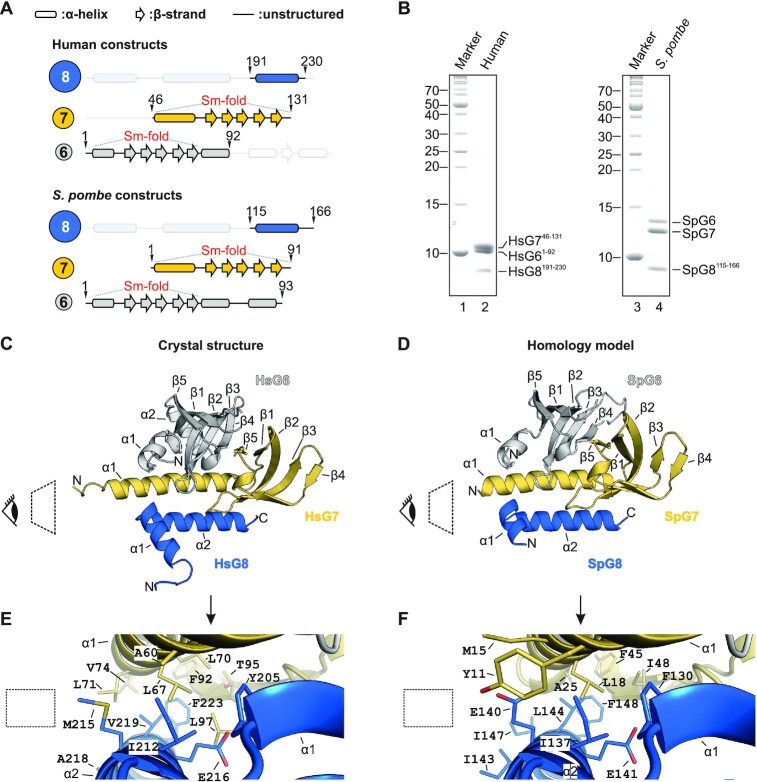
Crystal structure and homology model of the G6/G7/G8 module. (**A**) Representations of G8, G7 and G6 from human and *S. pombe* depicting the constructs used for purification, crystallization or homology modeling shown in B–F. (**B**) Gel filtration of HsG6^1–92^/HsG7^46–131^/HsG^191–230^ (1 and 2) and SpG6/SpG7/SpG8^115–166^ (3 and 4) complexes. (**C**) Crystal structure of HsG6^1–92^/HsG7^46–131^/HsG^191–230^. The C-terminus of HsG8 consisting of a helix (α1)-turn-helix (α2) motif engages with the N-terminal α1 helix of HsG7. The dimerization interface between HsG7 (β5) and HsG6 (β4) strands forming a continuous 10 sheet β-barrel remains intact. The respective N- and C- termini are labeled. (**D**) A homology model of SpG6/SpG7/SpG8^115–166^ generated using HsG6^1–92^/HsG7^46–131^/HsG^191–230^ crystal structure as a template. (**E**) Cluster of hydrophobic residues at the interface between HsG7 and HsG8. (**F**) Cluster of hydrophobic residues at the interface between SpG7 and SpG8. The homology model was generated using SWISS-MODEL and showed a QMEAN score of -1.4 with overall sequence identity of 24.44% between the template and the target sequences. Structures were generated using PyMOL Molecular Graphics System, Version 2.0, Schrödinger LLC.

We next focused on the structural investigation of the SMN complex. The structures of HsG6/G7 (47) and HsSMN/G2 modules are known (29,34) but the basis of G8-mediated bridging of both modules has not yet been established. We therefore expressed and purified complexes composed of the *S. pombe* proteins SpG6/SpG7/SpG8^115–166^ and the corresponding human proteins HsG6^1–92^/HsG7^46–131^/HsG8^190–230^, respectively (Figure 3A and B). The human complex allowed structure determination by X-ray crystallography and the generation of a homology model for the *S. pombe* orthologues (Figure 3C and D). The HsG6^1–92^/HsG7^46–131^/HsG8^190–230^ crystals yielded a 1.52 Å dataset and the structure was solved by molecular replacement using the HsG6/HsG7 structure (PDB ID: 1Y96). We obtained a complete atomic model (see Table 1 for crystallographic data and refinement statistics), which confirms the previously reported Sm-like fold of the HsG6/HsG7 dimer (47) and reveals the mode of HsG8 binding to HsG7. The C-terminus of HsG8 adopts a helix (α1)-turn-helix (α2) motif and interfaces with the N-terminal helix of HsG7 (Figure 3C). This interface comprises several highly conserved hydrophobic residues of HsG7 (A60, L67, L70, L71, F92 and L97) and of HsG8 (Y205, I212, M215, A218, V219 and F223) (Figure 3E). A hydrogen bond is established between the sidechain amino group of HsG7 Q56 and the carbonyl group of HsG8 R203. In addition, salt bridge interactions between the guanidine group of HsG7 R63 and sidechain carboxyl group of HsG8 E216 are also established (Supplementary Figure S5A). The corresponding *S. pombe* proteins SpG6, SpG7 and SpG8^115–166^ share 21%, 29% and 21% sequence identity, respectively, with their human counterparts. This allowed us to build a homology model of the *S. pombe* SpG6/SpG7/SpG8 complex based on our crystal structure (Figure 3D). The homology model showed that many conserved residues are clustered in the hydrophobic interface between SpG7 and SpG8 (Figure 3F, relevant residues are indicated). We therefore conclude that both systems possess a similar mode of interaction. Our results thus demonstrate a conserved modular architecture of the SpG6/SpG7/SpG8 sub-complex.

### Structural basis of SMN oligomerization

**Figure 4.**
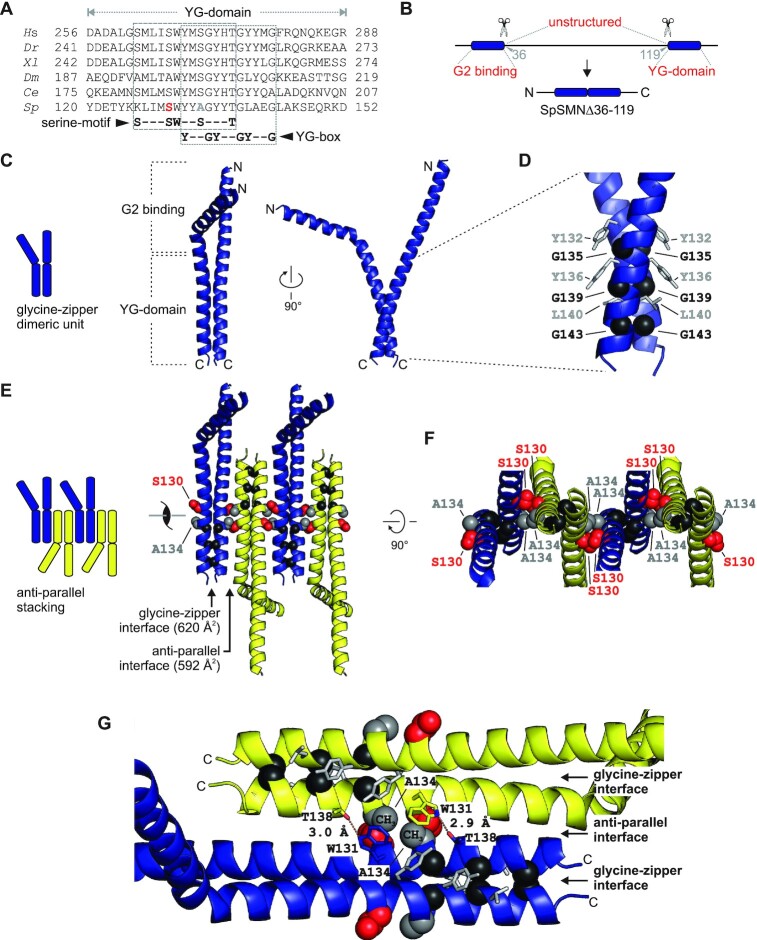
Crystal structure of SpSMN^Δ36–119^. (**A**) Multiple sequence alignment of SMN YG-domain of various organisms. Conserved motifs are highlighted with boxes. (**B**) Representative diagram showing generation of SpSMN^Δ36–119^ construct. (**C**) Crystal structure of SpSMN^Δ36–119^ showing YG-domain dimerization resulting in a glycine–zipper dimeric unit (the asymmetric unit). (**D**) Closeup view of the glycine–zipper dimeric unit showing interactions between YG-box residues of interfacing helices. The glycine residues are shown as black spheres. Tyrosine and Leucine residues are depicted as grey sticks. (**E**) Anti-parallel stacking of glycine–zipper dimeric units around a screw axis between S130 (red) and A134 (grey). Alternating dimeric units are colored yellow for clarity. The two distinct interfaces are indicated. (**F**) S130 and A134 of one helix pack tightly against A134 and S130, respectively, of the interfacing helix at the point of closest contact in the anti-parallel interface. The serine- and the alanine-sides alternate through consecutive anti-parallel interfaces. (**G**) Residue specific interactions on the alanine-side of the anti-parallel interface. Each W131 residue engages in hydrophobic interactions with the CH_3_ group of the interfacing A134. Sidechain conformation of each W131 is stabilized by H-bonding with T138 of the interfacing helix. Structures were generated using PyMOL Molecular Graphics System, Version 2.0, Schrödinger LLC.

We next investigated the oligomeric properties of the SpSMN complex. The C-terminal YG-domain of SMN is homologous across species with the two overlapping sequence elements, YxxGYxxGYxxG (YG-box) and SxxxSWxxSxxxT (serine-motif) being the key features (Figure 4A). The crystal structures of the human and *S. pombe* YG-domain had previously been solved (45,46) and revealed SMN dimerization via a glycine–zipper interaction of the YG-box. To re-evaluate this interaction, an SpSMN fragment lacking its unstructured middle region (SpSMNΔ36–119, Figure 4B) was co-expressed with a fragment of SpG2 lacking the N-terminus (SpG2ΔN80). The resulting SpSMN^Δ36–119^/SpG2^ΔN80^ complex was crystallized, a 2.16 Å dataset was collected and its structure solved by molecular replacement (Figure 4C–G) using the YG-domain fragment from the known MBP-SpYG-domain structure (PDB-ID:4RG5) (see Table 1 for crystallographic data and refinement statistics). We could detect clear electron density for SpSMN^Δ36–119^ but no electron density could be assigned to SpG2^ΔN80^, suggesting that the latter had dissociated and/or precipitated during crystallization. The structure revealed the SpSMN N-terminal G2 binding domain (residues 10–35) and the C-terminal YG-domain (residues 120–147) encompassing the YG-box and the serine-motif (Figure 4C). Two molecules of SpSMN^Δ36–119^ in the asymmetric unit (termed the glycine–zipper dimeric unit), interact via the YG-box residues of interfacing helices (Figure 4C). This interaction is identical to the previously observed interaction in the MBP-SpYG-domain crystal structure (PDB ID: 4RG5) and exhibits two sets of hydrophobic interactions. First, interfacing glycine residues (black spheres, Figure 4D) pack tightly against each other. Second, tyrosine and leucine residues of each helix (grey sticks, Figure 4D) pack tightly against glycine residues of the interfacing helix. Interestingly, a closer inspection of the crystallographic packing showed that each glycine–zipper dimeric unit is stacked upon each other in an anti-parallel fashion around a screw axis between S130 and A134, leading to an infinite stacking along the crystallographic A axis (Figure 4E and Supplementary Figure S5B). This interface, termed the anti-parallel interface, buries a surface area of 592 Å^2^ which is similar to the 620 Å^2^ buried surface area within the glycine–zipper interface (Figure 4E).

Anti-parallel stacking of glycine–zipper dimeric units is facilitated by the serine-motif where S130 and A134 pack against A134 and S130, respectively, of the interfacing helix of the adjacent dimeric unit, through mainchain atoms (Figure 4E and F). These reciprocal interactions place interfacing serine and alanine residues on opposite sides of the oligomeric stack (Figure 4F). As a consequence, the serine- and alanine-sides alternate through consecutive anti-parallel interfaces (Figure 4F). The alanine-side forms crucial interactions necessary for the formation of higher order oligomers (Figure 4G). The methyl group of each A134 forms hydrophobic contacts with the W131 sidechain of the interfacing helix. The sidechains of each W131 are stabilized by hydrogen bonding to the interfacing T138 sidechains (Figure 4G). As a result of these interactions, interfacing A134 residues remain fully buried at the center of the anti-parallel interface, while the S130 residues are only partially buried and therefore accessible for additional interactions.

### SMN oligomerization determines the SMN complex composition

**Figure 5.**
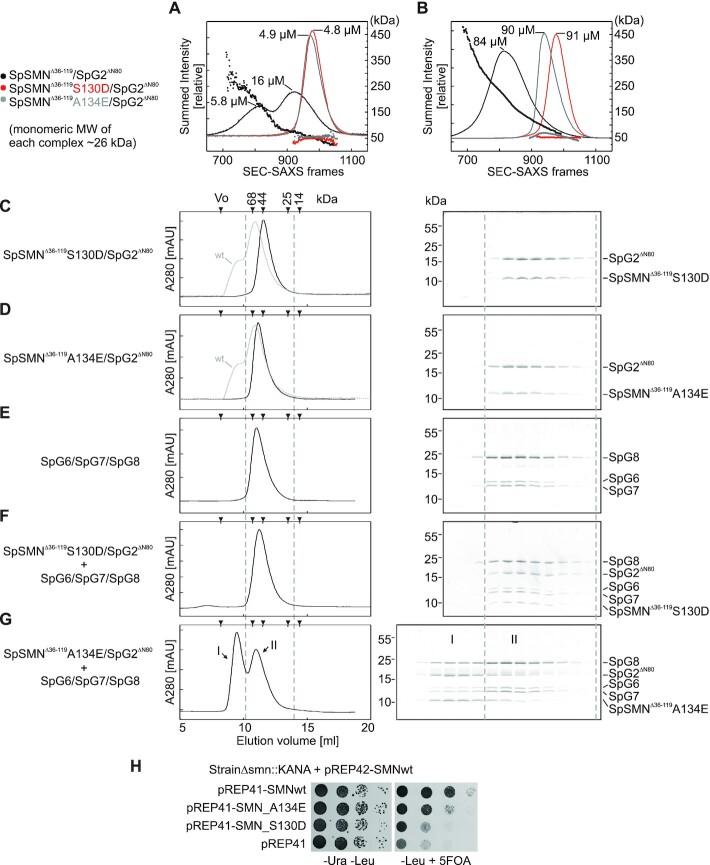
*In vitro* and *in vivo* analysis of anti-parallel interface through disruptive mutations. (**A** and **B**) Small angle X-ray scattering coupled to size exclusion chromatography (SEC-SAXS) chromatograms of indicated complexes at low (A) and high concentrations (B). The chromatograms are represented as (Summed Intensity vs SEC-SAXS frame number). Molecular weights for each frame within the chromatogram are shown as scatter plots. Peak concentrations for each chromatogram are indicated. (**C–E**) Control gel filtration runs (using Superdex 75 10/300) and SDS-PAGE analysis of SpSMN^Δ36–119^S130D/SpG2^ΔN80^ (C), SpSMN^Δ36–119^A134E/SpG2^ΔN80^ (D), and SpG6/SpG7/SpG8 (E). The wild-type (wt) complex (SpSMN^Δ36–119^/SpG2^ΔN80^) is shown as grey dotted chromatogram for comparison. (**F** and **G**) Complexation assay. SpG6/SpG7/SpG8 was mixed with equimolar amounts of either SpSMN^Δ36–119^S130D/SpG2^ΔN80^ (F) or SpSMN^Δ36–119^A134E/SpG2^ΔN80^ (G), and complex formation was monitored by gel filtration (using Superdex 75 10/300) and SDS-PAGE analysis. The A134E mutant forms a distinct pentameric complex with SpG6/SpG7/SpG8 (peak I). Excess SpG6/SpG7/SpG8 is separated in peak II. (**H**) Viability assay of full-length SpSMNwt and oligomerization defective mutants S130D and A134E. Yeast strain lacking endogenous SMN and carrying a plasmid containing the wild-type version of SpSMN and URA4 marker, was transfected with plasmids containing LEU2 marker with either SpSMNwt, SpSMN_A134E, SpSMN_S130D, or the empty vector. Yeast cells were spotted in 10-fold dilutions on (–Ura, –Leu) or on (–Leu, +5FOA) plates and incubated at 30°C.

A set of experiments was performed to test whether the newly discovered anti-parallel interface of SMN is physiologically relevant. We reasoned that residues with bulkier sidechains at this interface would prevent oligomer formation but would not impact the glycine–zipper interface. Hence, we substituted either S130 to aspartate (S130D) or A134 to glutamate (A134E) and analyzed the oligomeric states of the mutants by small angle X-ray scattering coupled to size exclusion chromatography (SEC-SAXS). Wild-type SpSMN^Δ36–119^/SpG2^ΔN80^ forms oligomers in the range of dimers to decamers at low concentrations (peak concentrations 5.8–16 μM, Figure 5A) but converts entirely to higher order oligomers when the concentration is increased (peak concentration 84 μM, Figure 5B). Both mutants, however, form exclusively dimers at any concentration (Figure 5A and B), but no higher order oligomers. This suggests that the anti-parallel interface is the major determinant for higher order oligomerization in solution but irrelevant for dimerization. Our results thus corroborate the previous notion that the SMN glycine–zipper dimers are the fundamental unit of higher order oligomers (46) and reveal a novel anti-parallel interface between glycine–zipper dimers required for higher order oligomer formation.

Next, we tested whether SMN oligomerization is relevant for the biochemical composition and/or function of the SMN complex. Based on our finding that SpSMN/G2 binds SpG6/SpG7/SpG8 and thus enables pentamer formation (Figure 2B), we asked whether the SMN mutants S130D and A134E can engage in similar interactions despite their oligomerization defect. To this end, we analyzed binding of the mutant dimers SpSMN^Δ36–119^S130D/SpG2^ΔN80^ and SpSMN^Δ36–119^A134E/SpG2^ΔN80^ to the trimeric module SpG6/SpG7/SpG8. SpSMN^Δ36–119^S130D/SpG2^ΔN80^ failed to form the pentameric SpSMN complex completely (compare Figure 5C, E and F). SpSMN^Δ36–119^A134E/SpG2^ΔN80^ in contrast, formed the pentameric SpSMN complex albeit with much lower efficiency as compared to the wild type (compare Figure 5D, E and G). Thus, mutations in the YG-domain that specifically interfere with SMN oligomerization but do not affect dimerization, compromise, or even prevent SMN complex formation *in vitro*.

Based on this observation we asked whether the mutations S130D and A134E in the YG-domain, impact on the viability of *S. pombe* (Figure 5H). For this, we generated a strain with a chromosomal deletion of *SMN* complemented by a pREP42 plasmid encoding the wild-type *SMN* gene and the *URA4* marker. The *SMN* mutants were subcloned into the pREP41 vector carrying a *LEU2* marker and their phenotypes were determined by spotting cells onto plates containing 5-fluoroorotic acid (5FOA). Since 5FOA selects cells that have lost the *URA4* plasmid, the phenotype of strains on this media will be due to the *SMN* mutant genes. Both mutants display a growth defect compared to the wild-type *SMN* gene (Figure 5H). The S130D mutant is more severe than the A134E mutant, which is consistent with our biochemical analysis. Together these results show that loss of SMN oligomerization impacts on yeast viability and is thus functionally relevant.

### SMA-causing mutations interfere with SMN oligomerization and SMN complex composition

**Figure 6.**
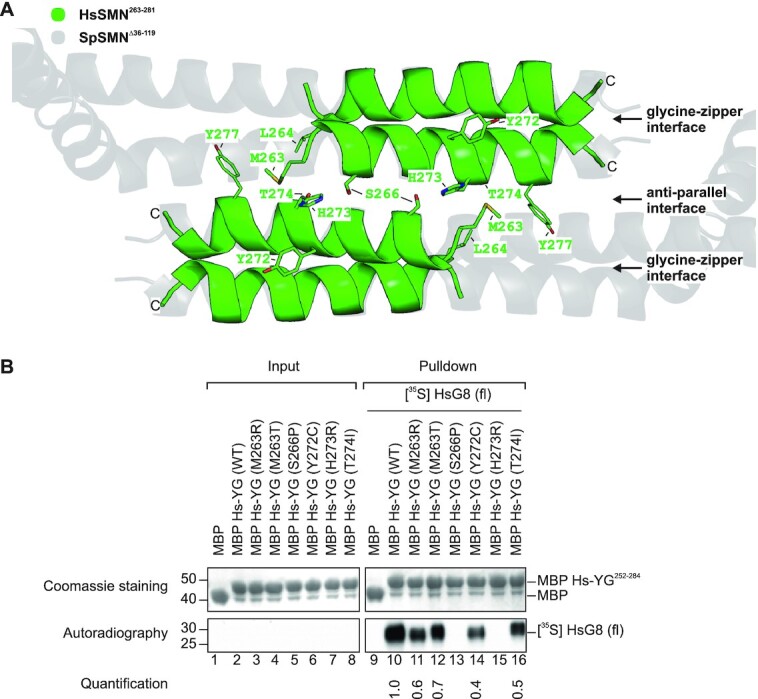
SMA missense mutations at the anti-parallel interface of human YG-domain tetramer. (**A**) Superimposition of the human YG-domain^263–281^ glycine–zipper dimeric units (green, PDB ID: 4GLI) onto the anti-parallel tetrameric SpSMN^Δ36–119^ structure (grey, this work). Residues implicated in SMA are depicted as sticks. (**B**) Pulldown assay of MBP-Hs-YG-domain^252–284^ (wt and SMA mutants). Immobilized MBP-Hs-YG-domain^252–284^ constructs were incubated with *in vitro* translated [^35^S]-labeled HsG8 (full-length). Eluates were analyzed by SDS-PAGE and Coomassie staining, and autoradiography. The relative quantifications of [^35^S]-labeled HsG8 from each IP are indicated below the respective lanes. Structural models were generated using PyMOL Molecular Graphics System, Version 2.0 Schrödinger, LLC.

The YG-domain of human SMN is a hotspot for missense mutations causing the motoneuron disease SMA. In fact, nearly 50% of known mutations are located in this region and have been shown to interfere with SMN oligomerization (45). We hence asked whether the oligomerization observed for the *S. pombe* YG-domain can also occur in human SMN and whether this is affected by SMA-causing missense mutations. To this end, we first constructed a model of the human YG-domain^263–281^. We used the reported structure of the human YG-domain^263–281^ fused to MBP (PDB ID: 4GLI), which only forms glycine–zipper dimers due to steric obstruction by the MBP fusion protein (45). In our model, we populated both interfaces by superposition of the human YG-domain^263–281^ structure onto the SpSMN^Δ36–119^ structure and energy minimized the final model (Figure 6A and Supplementary Figure S6). Of note, the human residues crucial for oligomer formation within the serine-motif (S266, W267, S270 and T274) are located precisely at positions relevant to establish a functional interface (compare Supplementary Figure S6B and C). The modeled human YG-domain^263–281^ structure is thus in perfect agreement with higher order oligomer formation as has been observed for the yeast system.

We then asked whether known SMA-causing missense mutations (65) would interfere with SMN oligomerization and/or G8 binding. To this end, we expressed MBP fused to the YG-domain^252–284^ containing SMA-causing missense mutations M263T, M263R, S266P, Y272C, H273R and T274I and analyzed their oligomeric properties by gel filtration chromatography (see Supplementary Table S2). With the exception of H273R, all missense mutations showed oligomerization defects to varying degrees. While M263R, S266P and Y272C existed predominantly as monomers, M263T and T274I existed as multiple oligomeric forms ranging from monomers to tetramers to octamers. A closer inspection of our tetrameric model of the human YG-domain^263–281^ shows that these residues are implicated in the glycine–zipper and/or the anti-parallel interface (Figure 6A and Supplementary Figure S6C). While S266 and T274 are crucial for the anti-parallel interface and are part of the serine-motif, Y272 is implicated in the glycine–zipper interface and is part of the YG-box. M263 on the other hand would form important hydrophobic interactions required for both interfaces (with L264 and Y277). Relative to these residues, H273 is oriented away from both interfaces and therefore does not show significant oligomerization defects compared to the wild-type construct. Hence, our anti-parallel oligomeric model of the human YG-domain^263–281^ supports the oligomerization defects observed for SMA missense mutations.

Next, binding of [^35^S]-labeled *in vitro* translated HsG8 to immobilized MBP fusion proteins of human YG-domain^252–284^ was analyzed. As shown in Figure 6B, M263R, M263T, Y272C and T274I show slightly reduced binding of HsG8 compared to the wild type. HsG8 binding to mutants S266P and H273R, on the other hand was entirely abolished. Since H273R does not show any oligomerization defects (see Supplementary Table S2), it stands to reason that residue H273 is part of an exposed surface required for HsG8 binding. Thus, pathogenic missense mutations cause specific defects in SMN oligomerization, which results in impaired binding of HsG8. Based on the critical role of G8 in the architecture of the SMN complex it is likely that this defect results in the loss of SMN complex integrity and function.

### SAXS analysis of SpSMN complex

With the characterization of the SpSMN complex, insight into its structural organization became feasible. We have determined the structural basis of SMN oligomerization via its YG-box and the serine motif, which showed anti-parallel multimerization of glycine–zipper dimeric units. As a consequence of this, the N-termini of SMN protrude on either side of the central oligomeric core. Such an arrangement would imply a high degree of disorder of the whole SMN complex and explain previously failed attempts to solve its structure by X-ray crystallography or cryo-EM. We therefore set out to use small angle X-ray scattering (SAXS) to generate additional data towards the goal of building a holistic model of the SMN complex. SAXS data provided various biophysical parameters of our complexes such as radius of gyration (*R*_g_), maximum particle dimension (*D*_max_), and molecular weight. In addition, dimensionless Kratky plots and pairwise distance distribution functions [*P*(*r*)] derived from SAXS data, illustrated the flexibility and disordered properties of the whole SpSMN complex.

**Figure 7.**
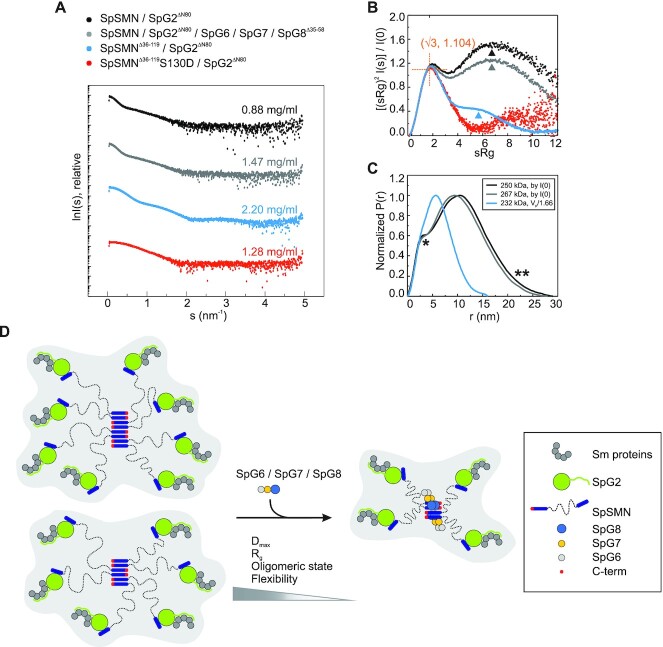
SAXS analysis and model of the SpSMN complex. (**A**) Small angle X-ray scattering curves of respective complexes at indicated concentrations represented as [I(s) vs s]. The scattering data have been deposited to SASBDB under the following accession codes: SASDKZ4, SASDK85, SASDK66, and SASDKF5. (**B**) Dimensionless Kratky plots [(sRg)^2^I(s)/I(0) versus sRg]. The expected maximum at (√3, 1.104) for globular entities is indicated by an orange crosshair. Deviation from globularity is indicated by arrowheads. (**C**) Normalized pairwise distance distribution functions represented as [Normalized P(r) vs r]. Molecular weights, either calculated from I(0) or from Porod volume (Vp/1.66) are indicated in the inset. For full length SpSMN complexes, * and ** represent shoulder and extended tail, respectively. (**D**) An integrative model of the SpSMN complex. The core of the SpSMN complex is formed by antiparallel multimerization (indicated by alternating SMN C-terminus) of glycine–zipper YG-domain dimeric units. The flexible N-terminal extensions of SpSMN (dotted lines) facilitate the capture of Sm proteins via the SpG2 subunit. The overall shape, flexibility, and oligomeric state of SpSMN is influenced by the SpG8/SpG7/SpG6 sub-complex.

We collected datasets of SAXS coupled to size exclusion chromatography (SEC-SAXS) for SpSMN/SpG2^ΔN80^, SpSMN/SpG2^ΔN80^/SpG6/SpG7/SpG8^Δ35–58^, SpSMN^Δ36–119^/SpG2^ΔN80^ and SpSMN^Δ36–119^S130D/SpG2^ΔN80^ (Figure 7A, see also Supplementary Table S3). Note that predicted unstructured regions of SpG2 (ΔN80) and SpG8 (Δ35–58) were deleted in these complexes (see also Figure 1A). The SpSMN^Δ36–119^S130D/SpG2^ΔN80^ complex was used in our analyses as a standard for globular entities (Figure 7B, see also Supplementary Table S3). Compared to the globular standard (red, Figure 7B), both complexes with full length SpSMN (black and grey, Figure 7B), exhibit dual behavior in the dimensionless Kratky plot. It shows a distinct maximum at the expected value for globular entities (66) (orange crosshair, Figure 7B), and a significantly raised signal at higher angles (black and grey arrowheads, Figure 7B), which is explained by the flexible region of SpSMN. The complex SpSMN^Δ36–119^/SpG2^ΔN80^ (with wild type YG-domain sequence, expected to form higher order oligomers) exhibited a shoulder (blue arrowhead, Figure 7B) typical for multi-domain proteins, but is highly compact in contrast to the full length SpSMN complexes. In addition, compared to the SpSMN^Δ36–119^/SpG2^ΔN80^ complex (232 kDa) (blue, Figure 7C), the normalized P(r) functions for full length SpSMN complexes of comparable molecular weights (black and grey, Figure 7C) showed asymmetric curves with a shoulder (*, Figure 7C) indicating multidomain architecture, and an extended tail region (**, Figure 7C) indicating disorder. These observations demonstrate that the SpSMN complex adopts highly extended conformations and behaves as a multidomain unit with flexible linkers. Interestingly, significant compaction was observed for the whole complex (SpSMN/SpG2^ΔN80^/SpG6/SpG7/SpG8^Δ35–58^) compared to SpSMN/SpG2^ΔN80^ (compare grey and black, Figure 7B), suggesting that additional factors control the conformation of SpSMN.

Next, using the data from standard, we determined the molecular weight, radius of gyration (R_g_) and maximum particle dimension (D_max_) for both full length SpSMN complexes at various concentrations (see Supplementary Table S3). SpSMN/SpG2^ΔN80^ was found to exist as a mixture of hexameric to octameric species. The oligomeric state of SpSMN/SpG2^ΔN80^/SpG6/SpG7/SpG8^Δ35–58^, however, is restricted to a tetrameric species at similar concentrations. In addition to this, both the R_g_ and the D_max_ are significantly reduced in the presence of SpG6/SpG7/SpG8^Δ35–58^. These results show that the oligomeric state and flexibility of the whole complex is influenced by the presence of the SpG6/SpG7/SpG8 module.

## DISCUSSION

The SMN complex of higher eukaryotes has been well studied at the biochemical and functional level and roles in several cellular pathways including the assembly of Sm-class U snRNPs, Pol II transcription and mRNP assembly/localization have been reported (6,67). Structural investigations of the SMN complex focused thus far only on smaller subunits linked to its role in UsnRNP assembly and revealed the basis of Sm-protein binding to SMN/G2 as well as the role of G5 in snRNA identification. Neither structures of higher order SMN oligomers, nor of the entire SMN complex have been determined. This is likely due to the fact that the unstructured regions of SMN, and of peripheral subunits are a major obstacle for conventional structure solution methods. The *S. pombe* system identified in this study combined with an integrative approach enabled the first structural investigation of the entire SMN complex. Detailed biochemical investigations first elucidated the overall architecture and interaction network of the entire complex (Figure 2). Using X-ray crystallography, in a second step, we were able to solve the structure of SpG6/SpG7/SpG8 as well the YG-domain oligomer, allowing homology modeling (Figures 3 and 4). Finally, using small angle X-ray scattering analysis we gained information about the SMN oligomerization behavior and its disordered regions which allowed the construction of an integrative model of the entire SMN complex (Figure 7).

The atomic structure of the C-terminal YG-domain of SpSMN reveals a structural key feature of the SMN complex. We discovered a novel interface that allows SMN glycine–zipper dimers to form higher order oligomers through an anti-parallel interaction interface via the serine-motif (SxxxSWxxSxxxT in higher eukaryotes and KxxxSWxxAxxxT in *S. pombe*). The second and third serine residues in this motif are located at the point of closest contact between two interacting glycine–zipper dimers at the anti-parallel interface (Figure 4E and F). Exceptions to the canonical motif are found in *D. melanogaster*, which has an alanine residue at the second serine position and *S. pombe*, which has an alanine at the third serine position (Figure 4A). Based on our oligomeric structure of the *S. pombe* YG-domain, these amino acid substitutions allow an anti-parallel stacking, and hence the formation of higher order oligomers. These findings are consistent with a previous study exploring alanine substitutions in the human YG-domain (45).

Several lines of evidence suggest that the anti-parallel interface is relevant for the integrity and function of the SMN complex: First, the SMN complexes reconstituted *in vitro* (this study) and detected *in vivo* (18,68) have hydrodynamic properties that are indicative of a multimeric rather than monomeric composition (Figure 2). Second, mutations in the anti-parallel interface preventing oligomerization display a growth defect compared to the wild-type SpSMN in *S. pombe*, with the phenotype being more severe upon loss of SpG6/SpG7/SpG8 binding (Figure 5). Third, these oligomerization defective mutants do not interfere with glycine–zipper dimerization (Figure 5). Fourth, the anti-parallel interaction surface is highly conserved between yeast and humans (Supplementary Figure S6). Furthermore, SMA-causing missense mutations not only affect dimerization but also oligomerization and impact on G8 binding (Figure 6 and Supplementary Table S2). Together, these results assign a novel function to the serine-motif residues of SMN.

The combination of X-ray crystallography and SAXS analysis allows us to propose a model for the architecture of the SpSMN complex, which likely also applies to its human counterpart (Figure 7D). The YG-domain of SpSMN nucleates the core of the complex and orchestrates its architecture. The existence of two independent interfaces in the YG-domain fosters the formation of higher order oligomers (Figure 4). This mechanism nucleates an interaction platform for the SpG6/SpG7/SpG8 module. Upon binding the overall shape of the SpSMN complex as well as its oligomeric state is strongly influenced, resulting in the predominant formation of a tetrameric SpSMN complex (see Supplementary Table S3). The SpG2 interacting N-termini of SpSMN protrude from the YG-domain oligomeric core and such arrangement might enable efficient capture of Sm proteins and their delivery onto UsnRNA during UsnRNP assembly.

A fundamental difference between SMN homologues of *S. pombe* and higher eukaryotes is the absence of the Tudor domain in the former (Figure 1A). We note that the anti-parallel arrangement of SMN subunits observed in our crystal structure is sterically favorable and thus allows the accommodation of Tudor domains within a fully assembled SMN complex. In addition, such a structural arrangement would also provide adequate space for additional factors that may interact with SMN such as profilin or Sm proteins. In contrast, a previously suggested parallel rotamer model of YG-domain (45) might be sterically unfavorable.

The YG-domain of SMN is a hotspot for SMA-causing missense mutations. The anti-parallel oligomer model of SMN reveals how these mutations affect the SMN complex and cause disease. We found that these mutations either interfere with the oligomerization state of SMN, G8 binding, or both. Whereas the majority of known missense mutations in the YG domain affect oligomerization only (Figure 6 and Supplementary Table S2) G8 binding was completely abolished by the two pathogenic missense mutations S266P and H273R. The former is associated with a strong oligomerization defect, which likely results from the disruption of the YG-domain helical structure. The latter, in contrast, does not display any oligomerization defect, suggesting that H273 might be directly involved in G8 binding. These observations are in line with the fact that both mutations, S266P and H273R, are associated with a type II manifestation of SMA, whereas T274I, which still allows G8 binding and displays only a minor reduction in its oligomeric state, leads to a milder (i.e. SMA type III) form of the disease (Figure 6 and Supplementary Table S2). We also observed that for some SMA-missense mutations, G8 binding is only partially affected despite the complete loss of SMN oligomerization (Figure 6 and Supplementary Table S2). This suggests that at least in vitro, monomeric YG-domain may be sufficient for G8 binding. Additional experiments such as the structural determination of the interaction of the YG-domain with G8 surface will uncover the precise relationship between SMN oligomerization and G8 binding.

Our studies also give a plausible explanation for a recently reported rescue experiment of *Smn* null mice with a combination of two pathogenic SMN missense mutations. It was shown that the combination of two SMN genes encoding the YG-domain mutant T274I and the Tudor domain mutant A111G can rescue *Smn* null mice when complemented in trans (69,70). Indeed, both mutants formed mixed SMN oligomers. This is in line with our model, which predicts the restoration of one of the two hydrogen bonds between W267 and T274 inside the anti-parallel interface (Supplementary Figure S6C). This likely stabilizes the chimeric SMN oligomer, thereby generating a functional SMN complex.

## DATA AVAILABILITY

HsG6^1–92^/HsG7^46–131^/HsG8^190–230^ and SpSMN^Δ36–119^ crystal structures have been deposited to Protein Data Bank (PDB) with accession codes 7BBL and 7BB3, respectively. SAXS data have been deposited to Small Angle Scattering Biological Data Bank (SASBDB) under the project https://www.sasbdb.org/project/1225/ with accession codes SASDKZ4, SASDK25, SASDK35, SASDK45, SASDK55, SASDK65, SASDK66, SASDK75, SASDK85, SASDK95, SASDKA5, SASDKB5, SASDKC5, SASDKD5, SASDKE5, and SASDKF5.

## Supplementary Material

gkab158_Supplemental_File
