# Supplementary material for: Identification and structural analysis of the Schizosaccharomyces pombe SMN complex
Source: Nucleic Acids Res. 2021 Mar 23;49(13):7207–23. doi: 10.1093/nar/gkab158 (PMC8287938; doi:10.1093/nar/gkab158)
Supplement: gkab158_Supplemental_File [file gkab158_supplemental_file.pdf]

Supplementary Figures and Tables

Identification and structural analysis of the *Schizosaccharomyces pombe* SMN complex

Jyotishman Veepaschit[1], Aravindan Viswanathan[1], Rémy Bordonné[2], Clemens Grimm[1*] and Utz Fischer[1*]

[1]Department of Biochemistry, Biocenter, University of Würzburg, Würzburg, 97074, Germany.

[2]Institut de Génétique Moléculaire de Montpellier, University of Montpellier, CNRS, Montpellier, 34293, France.

*To whom correspondence should be addressed: Utz Fischer

Tel: +49 931 31-84029

E-mail: utz.fischer@biozentrum.uni-wuerzburg.de

Correspondence may also be addressed to: Clemens Grimm

Tel: +49 931 31-84031

E-mail: clemens.grimm@biozentrum.uni-wuerzburg.de

Correspondence may also be addressed to: Rémy Bordonné

E-mail: Remy.Bordonne@igmm.cnrs.fr

Tel: +33 434 359647

The authors wished to be known that, in their opinion, the first 2 authors should be regarded as joint First Authors.

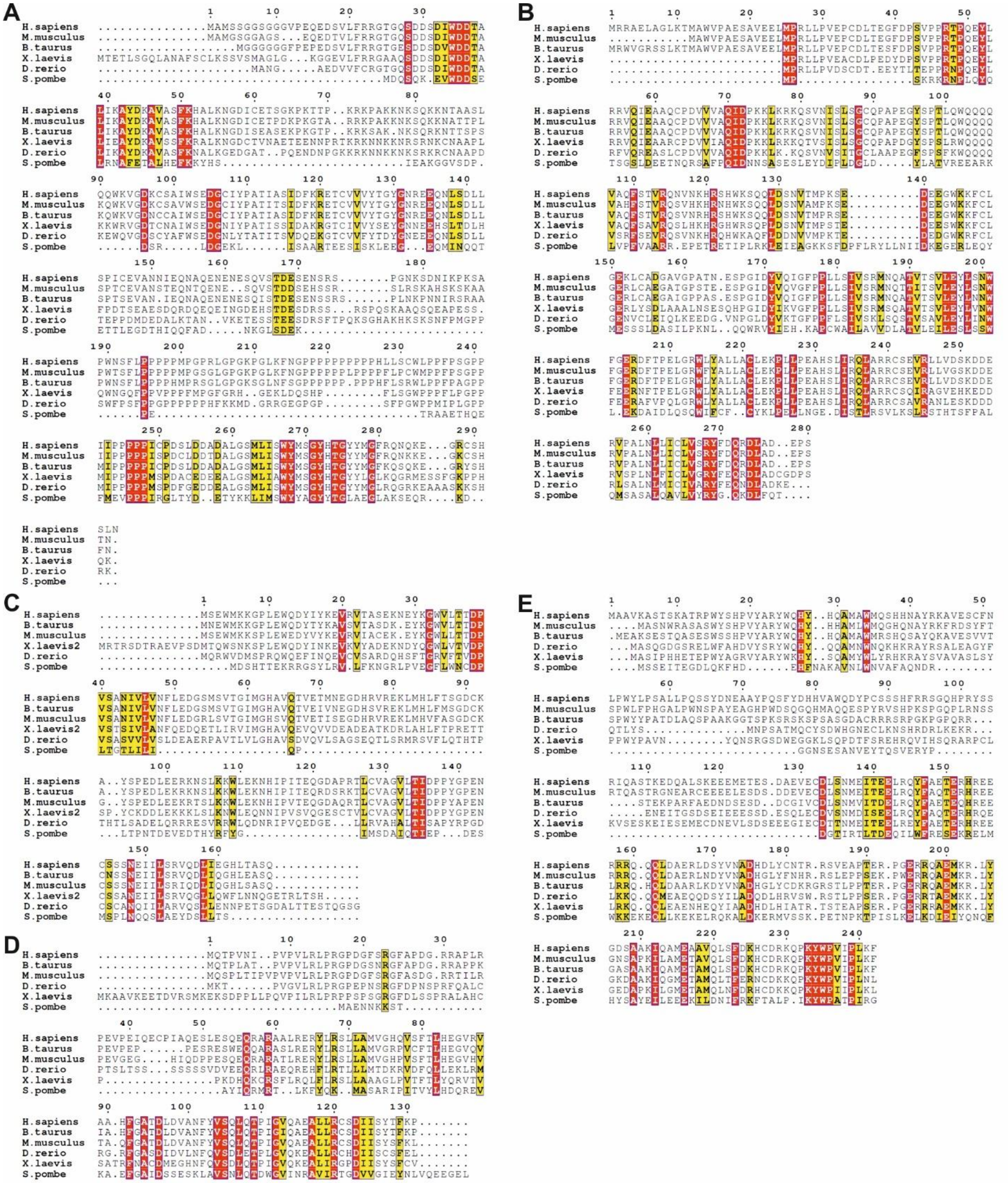

Supplementary Figure S1. Multiple sequence alignments of SMN complex components

Multiple sequence alignments of SMN (**A**), Gemin2 (**B**), Gemin6 (**C**), Gemin7 (**D**), and Gemin8 (**E**) from various organisms were performed using the ClustalW and ESPript webtools. Fully conserved amino acids are indicated in red, partially conserved amino acids are highlighted in yellow. The numbering are according to human proteins.

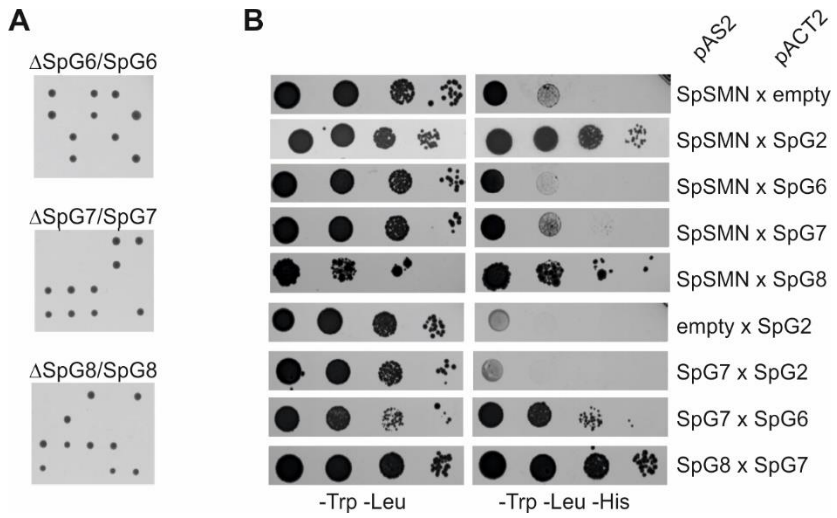

Supplementary Figure S2. In vivo analyses of SpGemins

(**A**) Tetrad analysis of heterozygous diploid S. pombe cells carrying a wild-type and a deleted copy of the indicated SpGemin gene. After sporulation, separated spores were grown on YES plates at 25°C for 5 days (the spores for each tetrad are arranged vertically). (**B**) Yeast two-hybrid interaction assays of SpGemins. The diploid reporter strain carrying yeast pAS2 and pACT2 two-hybrid plasmids expressing the indicated proteins was spotted in serial dilutions on –Leu–Trp or –Leu–Trp–His plates. Growth on –Leu–Trp–His plates is indicative of a positive interaction between two partners. Negative interactions are characterized by the absence of growth on –Leu–Trp–His plates. An empty vector served as a negative control.

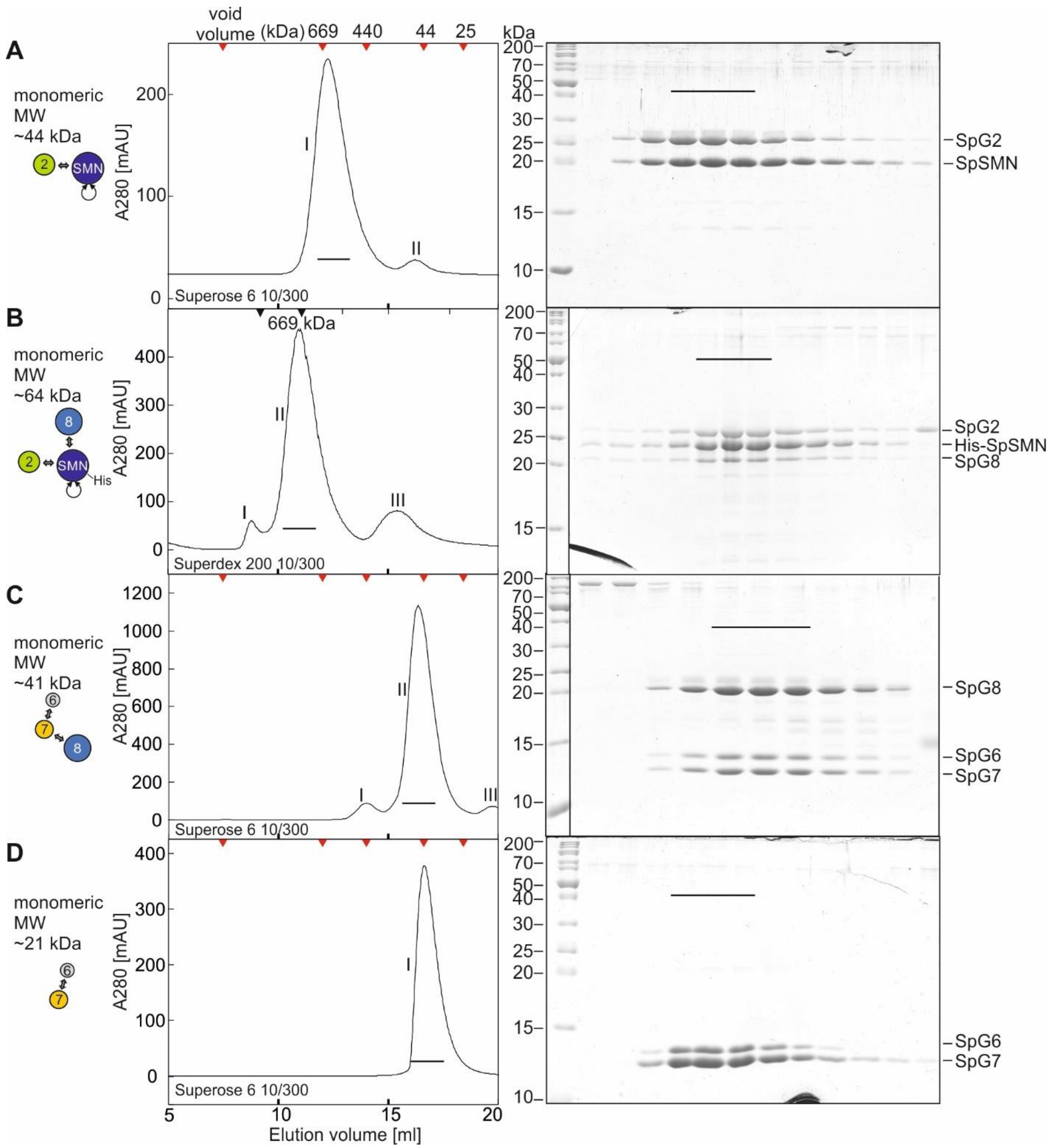

Supplementary Figure S3. Gelfiltration chromatography of SpSMN sub-complexes

SpSMN sub-complexes purified by Ni-NTA were analyzed by gelfiltration chromatography. (**A**) SpSMN/SpG2, (**B**) SpSMN/SpG2/SpG8, (**C**) SpG6/SpG7/SpG8, (**D**) SpG6/SpG7. Gelfiltration was performed using Superose 6 10/300 or Superdex 200 10/300 analytical columns (GE life sciences). Elution profiles with positions of standard molecular weight markers are shown above each chromatogram. Fractions were analyzed by 15% Tris-Tricine SDS-PAGE. Peak fractions are indicated by a black line. Monomeric molecular weight (MW) of each sub-complex is indicated above the representative diagram.

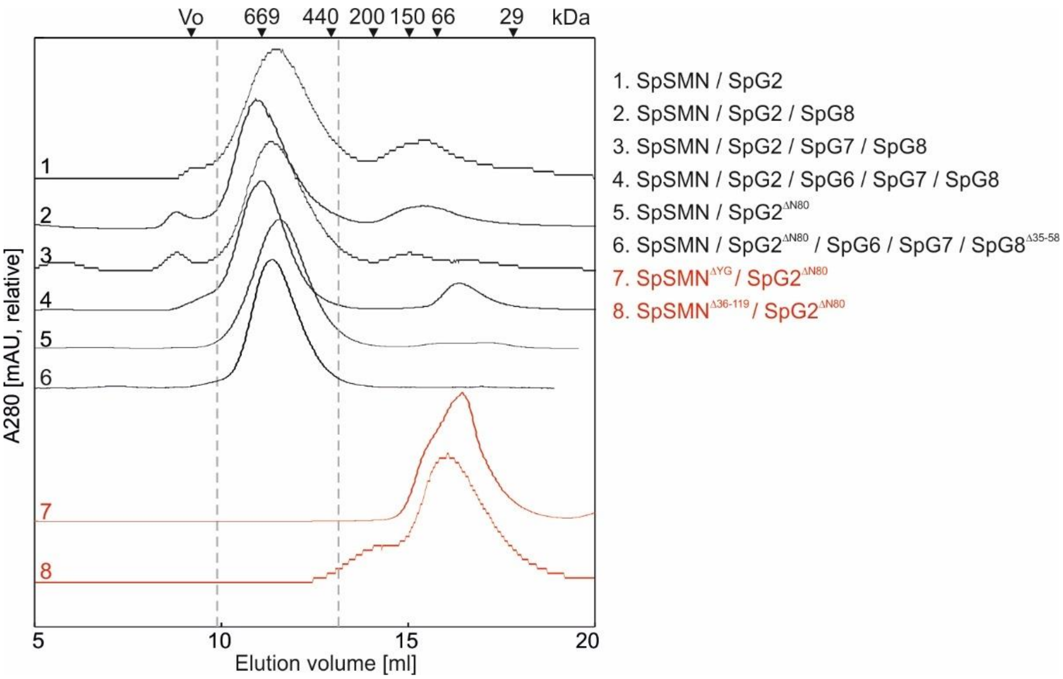

Supplementary Figure S4. Hydrodynamic properties of SpSMN complexes

Various SpSMN sub-complexes indicated in 1-8 were reconstituted in vitro and analyzed by gelfiltration (using Superdex 200 10/300, GE life sciences). All complexes containing full-length SpSMN exhibited almost identical size with a retention volume near the 669 kDa marker (within grey dashed lines). Flexible regions of SpG2 (residues 1-80) and SpG8 (residues 35-58) do not significantly contribute to the observed hydrodynamic sizes of the complexes (5 and 6). The complexes containing SpSMN lacking either the YG-domain (7) or the unstructured region (8, residues 36-119) showed drastic reduction in molecular sizes.

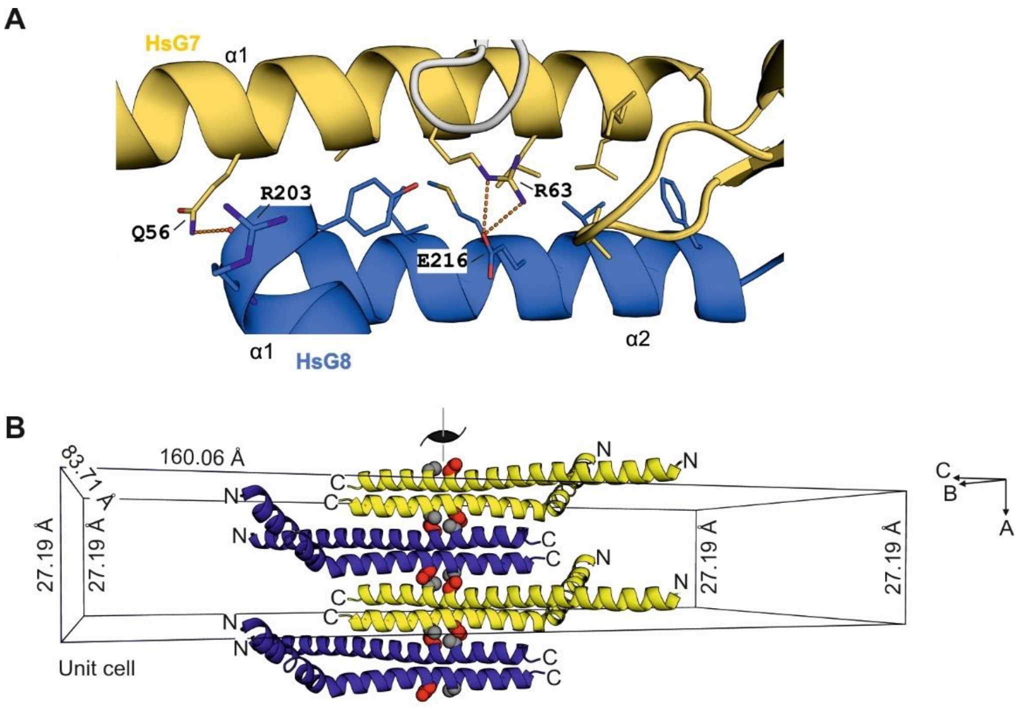

Supplementary Figure S5. Alternative views of crystal structures

(**A**) Specific atomic interactions between α1 of HsG7 and α1/2 of HsG8. Sidechain amino group of HsG7 Q56 establishes a hydrogen bond with the carbonyl group of HsG8 R203. In addition, salt bridge interactions between the guanidine group of HsG7 R63 and sidechain carboxyl group of HsG8 E216 are also evident. (**B**) Unit cell view showing the anti-parallel stacking of glycine-zipper dimeric units along the crystallographic axis A. Stacking of consecutive dimeric units occurs around a screw axis between S130 (red spheres) and A134 (grey spheres). The alternating N- and C-termini, and the unit cell dimensions are indicated. Structures were generated using PyMOL Molecular Graphics System, Version 2.0 Schrödinger, LLC.

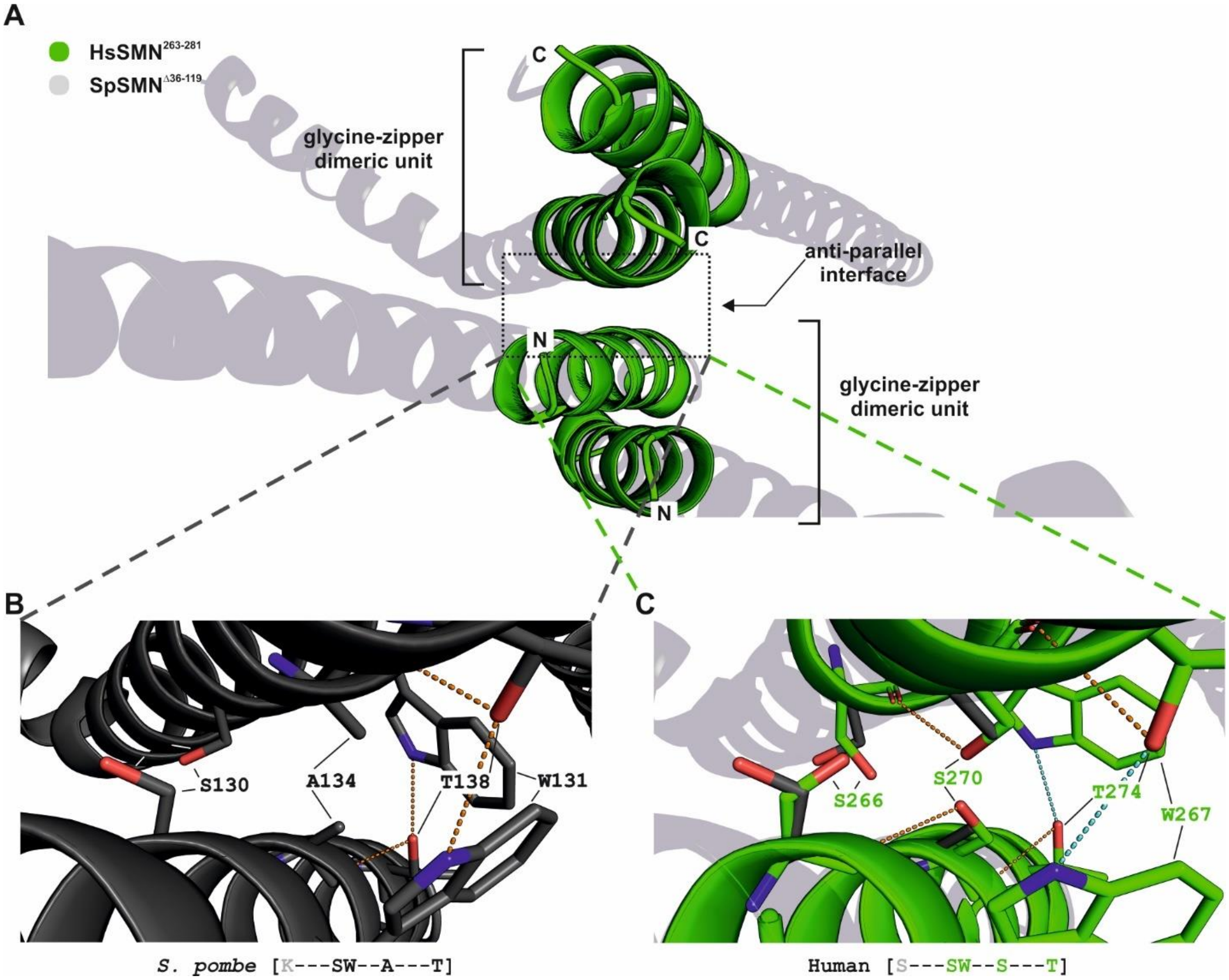

Supplementary Figure S6. Anti-parallel oligomeric model of human YG-domain glycine-zipper dimeric units

(**A**) Glycine-zipper dimeric units of the human YG-domain (green, PDB ID: 4GLI) are superimposed with the anti-parallel tetrameric SpSMN$^{\Delta 36\text{-}119}$ structure (grey, this work). The respective N- and C-termini are indicated. (**B** and **C**) Closeup views of the antiparallel interfaces of *S. pombe* (B) and human (C) systems showing key residues of the serine-motif engaging in various interactions. In the human system (C), the *S. pombe* residues S130 and A134 are also displayed (grey) for comparison. Note that an alternative conformation of S266 in the human system (as seen for *S. pombe* S130) would highly favor formation of the anti-parallel interface. Hydrogen bonds observed in crystal structures are depicted as orange dashed lines. Putative hydrogen bonds between interfacing W267 and T274 in human YG-domain tetrameric model are depicted as cyan dashed lines. Structures were generated using PyMOL Molecular Graphics System, Version 2.0 Schrödinger, LLC.

Supplementary Table S1. Mass-spectrometry analysis of GFP-SpG6 immunoprecipitation

GFP-SpG6 immunoprecipitate was subjected to mass spectrometry analysis. The number of identified peptides and coverage were compared with those of the control GFP-only immunoprecipitate. All five SpSMN complex components (SpSMN, SpG2, SpG6, SpG7, SpG8) as well as Sm proteins (SmD1, SmD2, SmD3, SmB, SmG, and SmF) were identified.

Identified protein name	MW (kDa)	Uniprot / PomBase ID	Nr. of Peptides identified		Coverage (%)	
			IP	*Control*	*IP*	*Control*
Gem-associated protein 7 homolog (SpG7)	10.336	G2TRR1 / SPBC32F12.16	10	2	87	25
Uncharacterized protein C16H5.15 (SpG8)	19.685	Q9USY8 / SPBC16H5.15	28	-	80	-
Survival motor neuron-like protein 1 (SpSMN)	17.385	Q09808 / SPAC2G11.08c	13	-	74	-
Uncharacterized protein new12 (SpG6)	10.623	G2TRK8 / SPAC4D7.15	3	-	74	-
Survival of motor neuron protein-interacting protein yip11 (SpG2)	26.994	P0CU08 / SPAC19B12.12c	15	-	46	-
Small nuclear ribonucleoprotein Sm D2	13.095	O14036 / SPAC2C4.03c	6	2	41	15
Small nuclear ribonucleoprotein-associated protein B	15.476	Q10163 / SPAC26A3.08	7	-	37	-
Small nuclear ribonucleoprotein Sm D1	13.089	O42661 / SPAC27D7.07c	3	-	30	-
Small nuclear ribonucleoprotein Sm D3	11.033	Q9UUC6 / SPBC19C2.14	3	1	25	7
Small nuclear ribonucleoprotein G	8.604	O74966 / SPBC4B4.05	2	-	22	-
Small nuclear ribonucleoprotein F	8.66	O59734 / SPBC3E7.14	1	-	13	-

Supplementary Table S2. Oligomeric states of MBP-HsYG-domain$^{252\text{-}284}$ SMA mutants

MBP-HsYG-domain$^{252\text{-}284}$ (wild-type or containing SMA missense mutations) was subjected to gelfiltration using Superdex 200 10/300 column (GE Life Sciences) and the resultant peak(s) were compared with the standard runs to estimate the oligomeric state.

Mutant	SMA type	Oligomeric status by gelfiltration
WT	Normal	Octameric
M263R	I	Monomeric
M263T	II	Tetramer-Monomer
S266P	II	Monomeric
Y272C	I	Monomeric
H273R	II	Octameric
T274I	III	Octamer-Tetramer-Monomer

Supplementary Table S3. SAXS derived biophysical parameters of SpSMN complexes

The zero angle intensity I(0) of SpSMN/SpG2$^{\Delta N80}$ and SpSMN/SpG2$^{\Delta N80}$/SpG6/SpG7/SpG8$^{\Delta 35\text{-}58}$ complexes, each at six different concentrations, were determined from the Guinier approximation of the scattering data. Since SpSMN$^{\Delta 36\text{-}119}$S130D/SpG2$^{\Delta N80}$ is a perfect dimer with a molecular weight of 52.5 kDa and shows highly globular properties (see Figure 7B), its concentration, I(0), and molecular weight were used as standard parameters, and the molecular weights of the afore mentioned complexes were calculated. In addition, the R_g and D_{max} of each complex was also determined (see Materials and Methods for further details).

**Complex**	**SASBDB Accession codes**	**MW$_{seq.}$†**	**Conc.**	**I(0)**	**MW$_{std.}$‡**	**Oligomeric state**	**R_g**	**D_{max}**
		kDa	*μM*	-	*kDa*		*nm*	*nm*
SpSMN$^{\Delta 36\text{-}119}$S130D/ SpG2$^{\Delta N80}$ (standard)	SASDKF5	26.25	49	27.21	52.5*	2-mer	2.7	9.0
SpSMN/SpG2$^{\Delta N80}$	SASDK75	35.47	3	10.27	237	6.7-mer	7.1	30.5
	SASDK25	35.47	4.6	18.78	281	7.9-mer	8.3	32
	SASDK65	35.47	5.2	18.81	251	7.1-mer	8.1	31
	SASDK35	35.47	11.8	45.28	267	7.5-mer	8.2	29.7
	SASDK55	35.47	11.9	41.28	241	6.8-mer	8.0	33
	SASDK45	35.47	24.8	87.77	247	6.9-mer	8.1	29
SpSMN/SpG2$^{\Delta N80}$/ SpG6/SpG7/ SpG8$^{\Delta 35\text{-}58}$/	SASDKE5	73.45	2.3	14.89	212	2.9-mer	6.3	21.3
	SASDK95	73.45	4.5	39.61	296	4.0-mer	8.0	29
	SASDKD5	73.45	5.1	38.48	255	3.5-mer	7.5	26
	SASDKA5	73.45	9.5	77.21	272	3.7-mer	8.0	27.3
	SASDKC5	73.45	9.7	71.13	247	3.4-mer	7.6	27
	SASDKB5	73.45	20	157.6	265	3.6-mer	7.9	28

† Molecular weight calculated from amino acid sequence of the monomeric complex

‡ Molecular weight calculated from I(0) using standard (see Materials and Methods for details)

* Known Molecular weight of the standard

Supplementary Table S4. SAXS data collection parameters

All small angle X-ray scattering data used in this work were collected coupled to size exclusion chromatography (SEC-SAXS) using a Superdex 200 10/300 column (GE life sciences) at the BM29 beam line of the ESRF storage ring (Grenoble, France).

Beamline	ESRF BM29 BioSAXS
Beam geometry	0.7 mm x 0.7 mm
Wavelength (Å)	0.9919
Sample-detector distance	2.867 m
Detector	Pilatus 1M
s-range ($Å^{-1}$)	0.0032 - 0.4944
Exposure time (sec)	1 per frame, 1800 frames
Concentration range	SEC-SAXS strategy (GE Superdex 200 10/300)
Temperature (K)	293
Primary data reduction	BM29 online data analysis, PRIMUS
1D data processing	ATSAS 3.0 package: CHROMIXS, PRIMUS, GNOM
3D graphics representations	PyMOL, Chimera
